# Mindfulness and self-compassion in trauma-related adjustment among adult women: a three-wave multi-regional study

**DOI:** 10.3389/fpsyg.2026.1716193

**Published:** 2026-05-29

**Authors:** Siqun Zhan, Dingfan Zhang, Yifan Ou

**Affiliations:** 1 Hubei Health Industry Development Research Center, School of Medical Humanities, Hubei University of Chinese Medicine, Wuhan, China; 2 Hubei Shizhen Laboratory, Wuhan, China; 3 College of Humanities and Management, Guilin Medical University, Guilin, Guangxi, China; 4 School of Social Sciences, University of Manchester, Manchester, United Kingdom; 5 Department of Communication Science, University of Antwerp, Antwerpen, Belgium

**Keywords:** adult women, depressive symptoms, dissociation, mindfulness, PTSD symptoms, self-compassion

## Abstract

Trauma-related distress in adult women often extends beyond posttraumatic stress disorder (PTSD) to include depressive symptoms and dissociation, yet the roles of mindfulness and self-compassion across these symptom domains remain insufficiently clarified. This study examined mindfulness and self-compassion as psychological resources associated with trauma-related symptoms among trauma-exposed adult women using a three-wave online survey design. The final sample included 240 women from eastern and western China. Mindfulness was operationalized through observation, acting with awareness, and non-reactivity, whereas self-compassion was measured through self-kindness, common humanity, and reduced self-criticism. Trauma-related distress was assessed through PTSD symptoms, depressive symptoms, dissociation, and an overall trauma symptom index. Results showed that mindfulness and self-compassion were negatively associated with all three symptom domains and independently predicted lower PTSD symptoms, depressive symptoms, dissociation, and overall trauma symptoms. Baseline mindfulness and self-compassion also significantly predicted lower subsequent symptom levels at T2. Regional differences were more evident in symptom burden than in psychological resources, with western participants reporting higher PTSD and overall trauma symptoms. Although trauma severity significantly interacted with both mindfulness and self-compassion, the interaction patterns did not support a classical buffering effect. Overall, mindfulness and self-compassion appear to be meaningful but context-dependent psychological resources in women's trauma-related adjustment.

## 1 Introduction

Trauma exposure remains a major mental health concern for adult women because trauma-related responses in women often extend beyond classic posttraumatic stress disorder (PTSD) symptoms to include depressive symptoms, dissociation, and broader disturbances in emotional functioning. Recent research has continued to show that post-trauma presentations are heterogeneous rather than uniform, and symptom burden may vary across trauma type, context, and co-occurring psychological difficulties. Contemporary evidence further suggests that women's trauma-related distress is frequently multidimensional, with PTSD often coexisting with depression and dissociative symptoms rather than emerging as an isolated syndrome (Hanáková et al., 2024; Fung et al., 2025). This broader perspective is particularly important in adult women, because trauma-related psychopathology in female samples has repeatedly been linked to emotional dysregulation, interpersonal adversity, and uneven post-trauma adaptation. At the same time, recent scholarship has increasingly emphasized the importance of identifying psychological resources that may be associated with lower trauma-related distress and more adaptive functioning over time. Among the most widely discussed candidates are mindfulness and self-compassion. A recent meta-analysis found that greater trait mindfulness was associated with fewer PTSD symptoms, while a more recent umbrella review concluded that mindfulness-based interventions show promising, although not uniformly consistent, effects for PTSD-related outcomes (Harper et al., 2022; Jovanovic and Garfin, 2024). Recent studies have likewise suggested that self-compassion may be relevant to trauma recovery, including prospective associations with posttraumatic responses and a potentially protective role in relation to trauma-related symptoms (Li et al., 2025; Adonis et al., 2025). In female trauma-exposed populations specifically, mindfulness- and compassion-oriented approaches have also shown promise in feasibility studies and intervention-oriented work, including studies involving women with high psychosocial burden (Braun et al., 2024; Garfin et al., 2025). Taken together, the recent literature suggests that mindfulness and self-compassion are not universal solutions, but they are theoretically and empirically plausible psychological resources that may be linked to lower trauma-related distress in adult women.

However, several important gaps remain in the current literature. First, many studies continue to focus primarily on PTSD as a single outcome, even though depressive symptoms and dissociation frequently co-occur with trauma-related distress and may reflect distinct but overlapping dimensions of post-trauma maladaptation. Second, much of the recent evidence remains cross-sectional, intervention-specific, or restricted to specialized samples, leaving comparatively fewer studies that examine whether baseline psychological resources are prospectively associated with multiple later trauma-related outcomes. Recent prospective work on self-compassion has begun to move in this direction, but the evidence is still limited and does not yet provide a sufficiently integrated account across multiple symptom domains (Li et al., 2025; Barel et al., 2025). Third, although contextual and cultural variation is increasingly acknowledged in trauma research, comparatively little work has directly examined whether associations among mindfulness, self-compassion, and trauma-related symptoms differ across regional subgroups within the same study. This limitation matters because symptom burden and adaptation may vary across settings even when the same general psychological resources are under consideration. Accordingly, the present study examines mindfulness and self-compassion as psychological resources associated with trauma-related symptoms among trauma-exposed adult women while simultaneously considering PTSD symptoms, depressive symptoms, dissociation, and an overall trauma symptom index. By adopting a three-wave, multi-regional design, the study seeks to clarify whether mindfulness and self-compassion are negatively associated with trauma-related symptoms at both the indicator and composite levels, whether baseline mindfulness and self-compassion prospectively predict lower subsequent symptom levels, and whether trauma-related symptoms differ across regional contexts. In doing so, the present study aims to provide a more empirically calibrated account of the role of mindfulness and self-compassion in women's trauma-related distress over time and across settings, rather than assuming in advance that these resources operate through a simple and uniform buffering pattern.

## 2 Literature review

### 2.1 Trauma-related symptoms in adult women

Trauma-related psychopathology in adult women should not be reduced to posttraumatic stress disorder (PTSD) alone, because accumulating evidence suggests that women's post-trauma responses are frequently multidimensional and characterized by substantial symptom overlap. In trauma-exposed women, particularly those with histories of interpersonal violence, PTSD commonly coexists with depressive symptoms, dissociative experiences, and broader emotional dysregulation, indicating that women's trauma burden is often better understood as a cluster of related but non-identical symptom domains rather than a single diagnostic outcome. Recent work has continued to document this complexity. A 2025 review of intimate partner violence and stress-related disorders emphasized that the long-term psychological consequences of trauma in women extend well beyond PTSD and frequently include depression and other chronic stress-related disturbances (Carannante et al., 2025). In a study of women who had experienced physical intimate partner violence, (Joseph et al. 2025) found that greater psychological difficulties were simultaneously reflected in posttraumatic stress, dissociative experiences, depression, and worry, underscoring the need to conceptualize women's trauma-related distress more broadly. Similarly, (Wang et al. 2023) argued that trauma-related depression should be treated as more than a secondary or peripheral issue in trauma research, because depression may emerge alongside PTSD and contribute to the persistence and complexity of post-trauma suffering. Dissociation is equally important in this regard. (Shim et al. 2024) showed that interpersonal trauma was related not only to depressive symptoms but also to dissociation, and further suggested that dissociation may serve as a pathway linking trauma to depression, especially in the context of childhood interpersonal trauma. Among women with co-occurring PTSD and substance use disorders, (Killeen and Brewerton 2023) found that the dissociative subtype was associated with greater PTSD severity and distinct clinical features, reinforcing the clinical relevance of dissociation in female trauma populations. Recent evidence also suggests that dissociation is not merely an accessory symptom but may mark a more severe post-trauma presentation. (Jarkas et al. 2025) reported that individuals with dissociative PTSD showed elevated PTSD and depression symptom severity relative to those with non-dissociative PTSD, while (Shelef et al. 2025) found that both depression and dissociation made significant contributions to PTSD severity. Treatment studies point in the same direction: (Sagcan et al. 2025) showed that symptoms linked to complex trauma in largely female clinical samples include not only core PTSD manifestations but also depression, dissociation, and broader disturbances in functioning, and (Gidzgier et al. 2023) further demonstrated that, in female patients with PTSD and substance use disorder, dissociation was tied to clinically serious outcomes and should not be ignored in trauma-related assessment. Taken together, these studies suggest that trauma-related symptoms in adult women are better conceptualized within a multi-outcome framework in which PTSD, depressive symptoms, and dissociation are simultaneously examined. This perspective provides a stronger conceptual basis for the present study, which does not restrict trauma-related distress to PTSD alone but instead treats PTSD symptoms, depressive symptoms, and dissociation as interconnected yet distinguishable manifestations of post-trauma maladjustment.

### 2.2 Mindfulness and self-compassion as psychological resources

Mindfulness and self-compassion have increasingly been conceptualized as two important psychological resources in trauma-related adjustment. In trauma research, mindfulness is commonly understood as a receptive and nonjudgmental awareness of present-moment experience, whereas self-compassion refers to responding to one's own suffering with kindness, reducing harsh self-criticism, and recognizing distress as part of the shared human condition. Although conceptually related, the two constructs are not identical: mindfulness is more directly concerned with how individuals attend to internal experience, whereas self-compassion is more closely tied to how they emotionally respond to themselves when confronted with pain, failure, or threat. This distinction is especially important in trauma contexts, because trauma survivors often struggle not only with intrusive memories and hyperarousal, but also with shame, self-blame, emotional avoidance, and difficulties in self-regulation. From this perspective, mindfulness may be relevant because it supports awareness without overidentification, while self-compassion may be relevant because it softens self-criticism and promotes a more accepting stance toward suffering. Recent trauma-sensitive work has increasingly supported this view. For example, qualitative research on a trauma-sensitive mindfulness and compassion group intervention found that participants described change in terms of empowerment, a new relationship to oneself and one's body, and greater freedom in relationships and life, suggesting that mindfulness- and compassion-based processes may be relevant to trauma recovery at the level of lived experience (Wästlund et al., 2024). More broadly, a 2025 randomized controlled trial of the Finding Solid Ground program reported improvements in PTSD symptoms, self-compassion, emotion regulation, and adaptive functioning among individuals with trauma-related dissociation, reinforcing the idea that self-compassion-related processes may be clinically meaningful in severe trauma presentations (Brand et al., 2025). ENT empirical studies have also strengthened interest in these constructs within trauma-exposed women and other high-burden populations. A randomized controlled study found that a mindfulness-based empowerment program enhanced self-compassion, self-esteem, and coping among women who had experienced violence, directly linking mindfulness-oriented intervention to self-compassion-related gains in a female trauma-exposed sample (Emirza and Bilgili, 2024). Similarly, adaptation work with trauma-exposed, unhoused women with substance use disorder has suggested that mindfulness-based interventions may be feasible and relevant even in highly disadvantaged settings characterized by severe trauma burden (Garfin et al., 2024). Qualitative evidence from women with comorbid PTSD and substance use disorder has further suggested that participants themselves perceive mindfulness-based interventions as working through mechanisms such as present-moment awareness, nonjudgmental acceptance, and self-compassionate attitudes toward suffering (Somohano et al., 2024). Outside exclusively female samples, recent trauma-informed mindfulness studies have also reported improvements in stress, mood, coping, compassion, self-compassion, and posttraumatic growth, indicating that these processes may support broader trauma recovery and resilience (Rousseau et al., 2024). In addition, a recent scoping review argued that nonattachment-related principles within mindfulness-based approaches may be relevant to post-traumatic stress recovery, further extending the theoretical rationale for examining mindfulness-related resources in trauma contexts (Tremblay et al., 2025). Finally, contemporary reviews of coping with racial trauma have also identified both mindfulness and self-compassion as evidence-based strategies with the potential to mitigate trauma-related psychological symptoms and foster empowerment, suggesting that their relevance is not confined to one single trauma context (Holmes et al., 2024). Taken together, the strongest conclusion supported by current evidence is not that mindfulness and self-compassion always buffer trauma in a simple or uniform way, but rather that they are promising psychological resources associated with more adaptive coping, better regulation, and lower trauma-related distress. This perspective provides the conceptual basis for examining mindfulness and self-compassion in relation to PTSD symptoms, depressive symptoms, and dissociation in the present study.

### 2.3 Gaps in longitudinal, multi-outcome, and contextual evidence

Despite growing interest in mindfulness and self-compassion in trauma-related research, several important gaps remain in the current literature. First, a substantial portion of the recent evidence is still cross-sectional, which limits stronger inferences about temporal ordering and makes it difficult to determine whether mindfulness and self-compassion precede later symptom differences or simply co-occur with them. Even when recent studies have moved beyond cross-sectional designs, longitudinal evidence remains comparatively limited and uneven across trauma contexts. For example, (Lorenzini et al. 2024) used a longitudinal, representative survey after collective trauma and found that trait mindfulness was associated with better subsequent mental health outcomes, but such designs remain relatively rare in the broader trauma literature. Likewise, recent work on self-compassion has increasingly suggested that self-related processes may matter over time, yet much of this evidence has focused on specific pathways or specialized samples rather than on broader multi-symptom prediction frameworks. For instance, (Peng and Ishak 2025) showed that self-compassion mediated links between insecure attachment and complex PTSD symptoms in college students with adverse childhood experiences, pointing to the temporal relevance of self-compassion but not fully addressing how mindfulness and self-compassion jointly relate to multiple later trauma-related outcomes in adult women. Overall, the literature still lacks sufficient longitudinal studies that simultaneously examine baseline psychological resources and later trauma-related symptoms across more than one symptom domain.

A second limitation is that many studies continue to focus primarily on PTSD as a single outcome, even though trauma-related distress often includes depressive symptoms, dissociation, shame-related processes, and broader self-functioning difficulties. Recent scholarship has increasingly highlighted this problem. (Harris et al. 2024), in a systematic review of mediators of complex trauma and complex post-traumatic stress disorder, identified multiple pathways beyond core PTSD symptoms, including dissociative processes, relationship-with-self variables, and emotional developmental processes. Similarly, (Morris et al. 2025) showed that self-compassion and trauma-related shame were both associated with health outcomes after interpersonal violence, suggesting that trauma-related adaptation is not adequately captured by PTSD severity alone. Related work on sexual trauma has also indicated that compassion-related variables may help explain broader global distress beyond a narrow PTSD framework (Dawood et al., 2024). These findings imply that studies restricted to PTSD alone may overlook important dimensions of trauma-related maladjustment, especially in women, whose post-trauma symptom profiles often include depression and dissociation alongside stress symptoms. A third limitation concerns contextual and regional evidence. Although recent studies increasingly acknowledge cultural and subgroup variation, comparatively little work has directly examined whether mindfulness- and compassion-related trauma processes differ across settings, regions, or sociocultural groups within the same design. (Morales and Burnett-Zeigler 2025) emphasized the need for culturally adapted mindfulness-based interventions, while (Reyes et al. 2025) reported racial differences in trauma-related outcomes after a mindfulness app intervention, suggesting that contextual variation may shape how mindfulness-related processes operate. In addition, recent trauma-sensitive mobile health work with forcibly displaced populations has underscored the importance of sociocultural adaptation and context-sensitive implementation in mindfulness- and compassion-based approaches (Zohar Puris et al., 2025). Taken together, the literature supports the relevance of mindfulness and self-compassion in trauma contexts, but it still leaves three key gaps insufficiently addressed: limited longitudinal evidence, inadequate multi-outcome modeling, and underdeveloped contextual or regional comparisons. These gaps provide a direct rationale for the present study, which uses a three-wave, multi-regional design to examine mindfulness and self-compassion in relation to PTSD symptoms, depressive symptoms, dissociation, and overall trauma symptoms among trauma-exposed adult women.

### 2.4 The present study

Against the above background, the present study examines mindfulness and self-compassion as psychological resources associated with trauma-related symptoms among trauma-exposed adult women. Rather than limiting trauma-related distress to PTSD alone, this study simultaneously considers PTSD symptoms, depressive symptoms, and dissociation, and further includes an overall trauma symptom index in order to capture the multidimensional nature of women's post-trauma adjustment more adequately. In addition, the study adopts a three-wave, multi-regional design to address the current lack of longitudinal, multi-outcome, and contextual evidence in this field. Specifically, the study has four aims. First, it examines whether mindfulness and self-compassion are negatively associated with trauma-related symptoms at both the indicator and composite levels. Second, it tests whether mindfulness and self-compassion independently predict PTSD symptoms, depressive symptoms, dissociation, and overall trauma symptoms in cross-sectional models. Third, it investigates whether baseline mindfulness and self-compassion prospectively predict lower subsequent symptom levels over time. Fourth, it explores whether key study variables differ across regional contexts. Based on the literature reviewed above, the study expects mindfulness and self-compassion to be associated with lower levels of trauma-related distress. At the same time, given the mixed and still limited evidence regarding contextual variation and interaction effects, the regional and moderation analyses are treated as exploratory rather than as tests of a fully established universal buffering model. In this way, the present study seeks to provide a more empirically calibrated account of how mindfulness and self-compassion relate to women's trauma-related symptoms across multiple domains, across time, and across settings.

## 3 Methods

### 3.1 Research design and participants

This study employed a three-wave online survey design to examine mindfulness and self-compassion as psychological resources among trauma-exposed adult women and to analyze their associations with trauma-related symptoms. Rather than focusing on a single trauma outcome, the present study simultaneously considered post-traumatic stress symptoms, depressive symptoms, and dissociation in order to provide a more comprehensive account of women's post-trauma psychological adjustment. In addition, a regional dimension was incorporated by comparing samples from eastern and western China, thereby allowing exploratory analysis of contextual differences in key variables.

Participants were adult women aged 18 years and older who had experienced at least one traumatic event. Trauma exposure included, but was not limited to, intimate partner violence, natural disasters, and accidents. Participants were recruited primarily through online channels, including social media platforms, mental health-related platforms, and cooperating organizations. The final valid sample consisted of 240 participants, including 120 from eastern China and 120 from western China. To ensure data quality, the inclusion criteria were as follows: participants had to be female, at least 18 years old, have a clear history of trauma exposure, and be able to complete the required measurements across the three study waves. Exclusion criteria included severe acute psychiatric crisis or failure to complete key assessment waves. The study included three measurement waves: T0 as the baseline assessment, T1 as the first follow-up, and T2 as the second follow-up. This design enabled the study to examine both cross-sectional associations among the variables and the predictive role of baseline psychological resources for subsequent symptom levels.

### 3.2 Measures

The study focused on three core constructs: mindfulness, self-compassion, and trauma symptoms. All indicators were measured on seven-point Likert-type scales, with higher scores indicating higher levels of the corresponding construct. For each construct, composite scores were calculated as the mean of the corresponding secondary indicators; therefore, composite values could take decimal forms. The indicator system, conceptual definitions, theoretical basis, and source instruments for all variables are summarized in Table 1.

**Table 1:** Indicator system.

Primary indicator	Secondary indicator	Definition	Theoretical basis	Data source
Mindfulness	Observation	Non-judgmental awareness of internal emotions, thoughts, and feelings.	Baer et al., 2006, five-facet model	FFMQ
Mindfulness	Acting with awareness	Deliberate engagement in behavior with present-moment awareness, avoiding automatic responses.	Shapiro et al., 2006, three-component model	FFMQ
Mindfulness	Non-reactivity	Maintaining balance when responding to negative emotions.	Baer et al., 2006, mechanisms of mindfulness	FFMQ
Self-compassion	Self-kindness	An accepting and warm attitude toward oneself in times of pain and failure.	Neff, 2003, theory of self-compassion	SCS-SF
Self-compassion	Common humanity	Viewing one's experiences as part of the shared human condition.	Neff, 2003, key components of self-compassion	SCS-SF
Self-compassion	Reduced self-criticism	Avoiding harsh self-judgment and showing self-care in suffering.	Neff, 2003, theory of self-compassion	SCS-SF
Trauma symptoms	PTSD symptoms	Symptoms of post-traumatic stress disorder, such as flashbacks and hyperarousal.	Brewin et al., 2009, PTSD symptomatology	PCL-5
Trauma symptoms	Depressive symptoms	Depressive mood following trauma, including anhedonia and persistent sadness.	Keng et al., 2011, post-trauma depression	PHQ-9
Trauma symptoms	Dissociation	Symptoms of dissociation after trauma, including depersonalization and derealization.	Brewin et al., 2009, trauma and dissociation	DES-II

#### 3.2.1 Mindfulness

Mindfulness was operationalized through three secondary indicators: observation, Acting with Awareness, and Non-reactivity. Observation refers to awareness of one's internal emotions, thoughts, and feelings. Acting with Awareness refers to deliberate engagement in behavior with present-moment attention rather than automatic responding. Non-reactivity refers to the tendency to maintain balance when encountering negative emotional experiences. The measurement framework was informed primarily by the Five Facet Mindfulness Questionnaire (FFMQ) and related theoretical work. The mean of the three indicators was computed as the composite mindfulness score, with higher values indicating greater mindfulness.

#### 3.2.2 Self-compassion

Self-compassion was measured through three secondary indicators: self-kindness, Common Humanity, and Reduced Self-criticism. Self-kindness refers to a warm and accepting attitude toward oneself in times of pain or failure. Common Humanity refers to viewing one's difficulties as part of the broader human experience. Reduced Self-criticism refers to a lower tendency to engage in harsh self-judgment or self-attack. The measurement approach was primarily informed by the Self-Compassion Scale-Short Form (SCS-SF) and its theoretical structure. Reduced Self-criticism was coded in the positive direction, such that higher scores reflected less self-criticism and greater self-compassion. The mean of the three indicators was used as the composite self-compassion score.

#### 3.2.3 Trauma symptoms

Trauma symptoms were represented by three secondary indicators: PTSD Symptoms, Depressive Symptoms, and Dissociation. PTSD Symptoms captured trauma-related stress reactions such as intrusion and hyperarousal. Depressive Symptoms reflected persistent low mood, anhedonia, and related depressive distress following trauma. Dissociation reflected symptoms such as depersonalization, derealization, and psychological detachment. These indicators were conceptually informed by the PCL-5, PHQ-9, and DES-II frameworks, respectively. The mean of the three indicators was computed as the overall trauma symptom score, with higher values indicating greater trauma-related distress.

### 3.3 Procedure

Data were collected through online survey platforms. At baseline (T0), participants completed the first wave of questionnaires, which included measures of mindfulness, self-compassion, trauma exposure, and trauma-related symptoms. The same general domains were then assessed again at the two follow-up waves in order to examine the relationships among the key variables over time. Survey links were distributed electronically at each wave, and reminder notifications were used to reduce participant attrition. All data were stored anonymously in encrypted environments to ensure privacy and data security. For regional comparison, participants were categorized into eastern and western subsamples based on their reported region of residence. This grouping was used for subsequent independent-samples comparisons and was intended to examine mean-level contextual differences rather than to assume fundamentally different psychological mechanisms across regions.

### 3.4 Data analysis

Although data were collected across three waves (T0, T1, and T2), the principal longitudinal analyses in the present study focused primarily on the predictive relationships between baseline psychological resources measured at T0 and subsequent trauma-related symptoms measured at T2. The T1 assessment was retained as part of the broader longitudinal design to support temporal continuity and participant retention across study waves. Data analysis proceeded in four steps. First, descriptive statistics were calculated to summarize the distribution of the secondary indicators and the core composite variables, including means, standard deviations, minimum values, and maximum values. Second, the reliability and validity of the measurement model were evaluated. Internal consistency reliability was assessed using Cronbach's alpha. Convergent validity was examined through standardized factor loadings, composite reliability (CR), and average variance extracted (AVE). Discriminant validity was assessed using the Fornell–Larcker criterion by comparing the square root of each construct's AVE with the inter-construct correlations. Third, cross-sectional relationships among the variables were examined. Pearson correlation coefficients were calculated to test the direction and strength of the associations among mindfulness, self-compassion, and trauma-related symptoms. Multiple linear regression models were then estimated with PTSD symptoms, depressive symptoms, dissociation, and overall trauma symptoms as dependent variables, and mindfulness and self-compassion as predictors, in order to test the independent predictive roles of these two psychological resources. Fourth, several additional analyses were conducted. Moderation models were estimated by including trauma severity, mindfulness or self-compassion, and their interaction terms to examine whether the relationship between trauma severity and PTSD symptoms varied across levels of psychological resources. Independent-samples *t*-tests were conducted to compare eastern and western participants on key variables. Finally, longitudinal regression models were used to test whether baseline mindfulness and baseline self-compassion predicted trauma-related symptoms at T2, thereby evaluating the prospective associations of these resources.

All analyses were conducted in a Python-based statistical environment. Data management was performed using pandas, significance testing and correlations were conducted using SciPy, and linear regression analyses were performed using statsmodels. Statistical significance was evaluated at the conventional thresholds of *p* < 0.05, *p* < 0.01, and *p* < 0.001.

### 3.5 Hypotheses

Building on the preceding theoretical review and the empirical gaps identified above, the present study develops a structured set of hypotheses concerning the relationships among mindfulness, self-compassion, and trauma-related symptomatology in trauma-exposed adult women. Specifically, the study proposes that mindfulness and self-compassion function as protective psychological resources that are associated with lower levels of PTSD symptoms, depressive symptoms, and dissociation, both concurrently and over time.

**H1. Negative associations hypothesis**: mindfulness and self-compassion will be negatively associated with trauma-related symptoms among trauma-exposed adult women. Specifically, higher levels of mindfulness and self-compassion will be associated with lower levels of PTSD symptoms, depressive symptoms, and dissociation.

**H2. Independent predictive effects hypothesis**: mindfulness and self-compassion will independently and negatively predict trauma-related symptoms. Specifically, both variables will show significant negative effects on PTSD symptoms, depressive symptoms, dissociation, and overall trauma symptoms in cross-sectional regression models.

**H3. Longitudinal predictive effects hypothesis**: baseline mindfulness and baseline self-compassion will prospectively predict lower subsequent trauma-related symptoms. Specifically, higher levels of mindfulness and self-compassion at T0 will be associated with lower levels of PTSD symptoms, depressive symptoms, and dissociation at later measurement points.

**H4. Regional difference hypothesis**: trauma-related symptoms will differ across regional groups. Specifically, participants from different regions will show significant differences in trauma-related symptom levels, whereas regional differences in mindfulness and self-compassion may be smaller or less consistent.

## 4 Empirical results

### 4.1 Descriptive statistics

Table 2 presents the descriptive statistics of the key variables included in this study. Overall, the mean scores of the mindfulness-related indicators were all above the midpoint of the seven-point scale, with Observation (*M* = 4.12, SD = 1.98), Acting with Awareness (*M* = 4.17, SD = 1.96), and Non-reactivity (*M* = 4.10, SD = 1.92) suggesting that the participants, on average, demonstrated a moderate level of mindfulness. Similarly, the self-compassion indicators also showed moderate average levels, including Self-Kindness (*M* = 3.97, SD = 1.93), Common Humanity (*M* = 3.98, SD = 1.87), and Reduced Self-Criticism (*M* = 4.00, SD = 1.91). In comparison, the trauma-related symptom indicators displayed somewhat varied but still moderate levels, with PTSD Symptoms (*M* = 3.35, SD = 1.98), Depressive Symptoms (*M* = 3.76, SD = 2.06), and Dissociation (*M* = 3.95, SD = 1.97). Among these, dissociation had the highest mean, followed by depressive symptoms, while PTSD symptoms showed the lowest average level. In addition, the standard deviations ranged from 1.87 to 2.06, indicating considerable individual variation across all variables. The full range of responses from 1 to 7 was observed for every indicator, suggesting that the sample captured substantial heterogeneity in participants' psychological resources and trauma-related experiences. Taken together, these results indicate that the sample exhibited moderate levels of mindfulness, self-compassion, and trauma symptoms overall, while also showing sufficient variability to support subsequent correlational and regression analyses.

**Table 2:** Descriptive statistics of key variables.

Variable	Mean	SD	Min	Max
Observation	4.12	1.98	1	7
Acting with awareness	4.17	1.96	1	7
Non reactivity	4.10	1.92	1	7
Self kindness	3.97	1.93	1	7
Common humanity	3.98	1.87	1	7
Reduced self criticism	4.00	1.91	1	7
PTSD symptoms	3.35	1.98	1	7
Depressive symptoms	3.76	2.06	1	7
Dissociation	3.95	1.97	1	7

Table 3 presents the descriptive statistics of the composite scores for the three core constructs examined in this study. Overall, participants reported a moderate level of mindfulness (*M* = 4.13, SD = 1.64) and self-compassion (*M* = 3.98, SD = 1.60) on the seven-point scale. The mean score for trauma symptoms was 3.68 (SD = 1.67), indicating that trauma-related distress was also present at a moderate level in the sample. Comparatively, mindfulness showed the highest mean among the three composite variables, followed by self-compassion, whereas trauma symptoms were slightly lower but still close to the midpoint of the scale. The standard deviations, ranging from 1.60 to 1.67, suggest substantial interindividual variation in all three constructs. In addition, the minimum and maximum values spanned the full possible range from 1.00 to 7.00 for each composite score, indicating that the sample captured considerable heterogeneity in participants' psychological resources and trauma-related experiences. Taken together, these findings suggest that the participants demonstrated moderate overall levels of mindfulness, self-compassion, and trauma symptoms, while also providing sufficient variability for subsequent correlational, regression, and longitudinal analyses.

**Table 3:** Descriptive statistics of composite scores.

Variable	Mean	SD	Min	Max
Mindfulness	4.13	1.64	1.00	7.00
Self compassion	3.98	1.60	1.00	7.00
Trauma symptoms	3.68	1.67	1.00	7.00

### 4.2 Reliability, convergent validity, and discriminant validity

Table 4 reports the reliability and convergent validity results for the measurement model. First, all standardized factor loadings were high, ranging from 0.81 to 0.87, indicating that each observed indicator loaded strongly on its intended latent construct. Specifically, the three indicators of mindfulness showed loadings between 0.81 and 0.84, the self-compassion indicators ranged from 0.82 to 0.85, and the trauma symptom indicators ranged from 0.84 to 0.87. These results suggest that the indicators demonstrated satisfactory item reliability. Second, the Cronbach's alpha coefficients for the three constructs were 0.79 for mindfulness, 0.79 for self-compassion, and 0.80 for trauma symptoms, all of which exceeded the commonly accepted threshold of 0.70, supporting adequate internal consistency reliability. Third, the composite reliability (CR) values ranged from 0.87 to 0.89, further indicating strong construct reliability. Finally, the average variance extracted (AVE) values ranged from 0.68 to 0.73, all above the recommended cutoff of 0.50, which supports good convergent validity. Taken together, these results indicate that the measurement model demonstrated satisfactory reliability and convergent validity, suggesting that the three latent constructs were measured with acceptable consistency and precision.

**Table 4:** Reliability and convergent validity of the measurement model.

Construct	Indicator	Standardized loading	Cronbach's α	CR	AVE
Mindfulness	Observation	0.81	0.79	0.87	0.68
Mindfulness	Acting with awareness	0.84			
Mindfulness	Non-reactivity	0.83			
Self compassion	Self-kindness	0.82	0.79	0.88	0.70
Self compassion	Common humanity	0.84			
Self compassion	Reduced self-criticism	0.85			
Trauma symptoms	PTSD symptoms	0.87	0.80	0.89	0.73
Trauma symptoms	Depressive symptoms	0.84			
Trauma symptoms	Dissociation	0.86			

Table 5 presents the discriminant validity results based on the Fornell–Larcker criterion. The square roots of the average variance extracted (AVE), shown on the diagonal, were 0.827 for mindfulness, 0.837 for self-compassion, and 0.857 for trauma symptoms. In each case, the diagonal value was greater than the corresponding inter-construct correlations. Specifically, the correlation between mindfulness and self-compassion was 0.421, whereas the correlations of trauma symptoms with mindfulness and self-compassion were −0.378 and −0.344, respectively. Because the square root of the AVE for each construct exceeded its correlations with the other constructs, the results support satisfactory discriminant validity. In other words, although mindfulness, self-compassion, and trauma symptoms were meaningfully related, they remained empirically distinguishable from one another. These findings indicate that the three latent constructs captured conceptually related but statistically distinct dimensions, thereby supporting the appropriateness of retaining them as separate variables in the subsequent analyses.

**Table 5:** Discriminant validity based on the Fornell–Larcker criterion.

Construct	Mindfulness	Self compassion	Trauma symptoms
Mindfulness	0.827		
Self compassion	0.421	0.837	
Trauma symptoms	−0.378	−0.344	0.857

### 4.3 Correlation analysis

Table 6 presents the correlation matrix of the secondary indicators. Overall, the three mindfulness indicators were positively and significantly correlated with one another, with coefficients ranging from 0.49 to 0.63 (all *p* < 0.001), indicating a coherent internal structure of the mindfulness construct. Similarly, the self-compassion indicators were also positively interrelated. Self-kindness was significantly associated with common humanity (*r* = 0.54, *p* < 0.001) and reduced self-criticism (*r* = 0.63, *p* < 0.001), while common humanity was positively correlated with reduced self-criticism (*r* = 0.49, *p* < 0.001). These results support the internal consistency of the self-compassion dimension. In addition, positive correlations were observed between the mindfulness and self-compassion indicators, with coefficients ranging from 0.19 to 0.40, suggesting that these two sets of psychological resources were related but not redundant. By contrast, all mindfulness and self-compassion indicators were negatively correlated with the trauma-related symptom indicators. Specifically, PTSD symptoms showed significant negative correlations with observation (*r* = −0.20, *p* < 0.01), acting with awareness (*r* = −0.24, *p* < 0.001), non-reactivity (*r* = −0.22, *p* < 0.001), self-kindness (*r* = −0.24, *p* < 0.001), common humanity (*r* = −0.24, *p* < 0.001), and reduced self-criticism (*r* = −0.18, *p* < 0.01). A similar pattern was found for depressive symptoms and dissociation, both of which were negatively associated with all or nearly all of the psychological resource indicators. Among the symptom variables themselves, PTSD symptoms were positively correlated with depressive symptoms (*r* = 0.59, *p* < 0.001) and dissociation (*r* = 0.54, *p* < 0.001), while depressive symptoms and dissociation were also strongly positively related (*r* = 0.59, *p* < 0.001). Taken together, these findings indicate that higher levels of mindfulness and self-compassion at the indicator level were associated with lower levels of trauma-related symptoms, whereas the symptom indicators were moderately to strongly interrelated with one another. This overall pattern is theoretically consistent and provides preliminary support for the hypothesized relationships among the study variables.

**Table 6:** Correlation matrix of secondary indicators.

Variable	1	2	3	4	5	6	7	8	9
Observation	1								
Acting with awareness	0.63^***^	1							
Non reactivity	0.49^***^	0.55^***^	1						
Self kindness	0.34^***^	0.36^***^	0.24^***^	1					
Common humanity	0.31^***^	0.40^***^	0.28^***^	0.54^***^	1				
Reduced self criticism	0.26^***^	0.30^***^	0.19^**^	0.63^***^	0.49^***^	1			
PTSD symptoms	−0.20^**^	−0.24^***^	−0.22^***^	−0.24^***^	−0.24^***^	−0.18^**^	1		
Depressive symptoms	−0.33^***^	−0.37^***^	−0.35^***^	−0.34^***^	−0.28^***^	−0.25^***^	0.59^***^	1	
Dissociation	−0.18^**^	−0.21^**^	−0.25^***^	−0.26^***^	−0.16^*^	−0.21^**^	0.54^***^	0.59^***^	1

^***^Indicates statistical significance at the *p* < 0.001 level; ^**^Indicates statistical significance at the *p* < 0.01 level; ^*^Indicates statistical significance at the *p* < 0.05 level.

Table 7 presents the correlation matrix of the composite scores for the main study variables. Mindfulness was positively and significantly correlated with self-compassion (*r* = 0.42, *p* < 0.001), indicating that individuals with higher levels of mindfulness also tended to report greater self-compassion. In contrast, both mindfulness and self-compassion were negatively associated with the three trauma-related symptom variables. Specifically, mindfulness was negatively correlated with PTSD symptoms (*r* = −0.27, *p* < 0.001), depressive symptoms (*r* = −0.42, *p* < 0.001), and dissociation (*r* = −0.26, *p* < 0.001). Similarly, self-compassion showed significant negative correlations with PTSD symptoms (*r* = −0.28, *p* < 0.001), depressive symptoms (*r* = −0.35, *p* < 0.001), and dissociation (*r* = −0.25, *p* < 0.001). These results suggest that higher levels of mindfulness and self-compassion were associated with lower levels of trauma-related distress. In addition, the trauma symptom variables were positively interrelated. PTSD symptoms were significantly correlated with depressive symptoms (*r* = 0.59, *p* < 0.001) and dissociation (*r* = 0.54, *p* < 0.001), while depressive symptoms were also positively correlated with dissociation (*r* = 0.59, *p* < 0.001). Overall, the pattern of associations was theoretically consistent, indicating that mindfulness and self-compassion functioned as protective psychological resources, whereas PTSD symptoms, depressive symptoms, and dissociation clustered together as related manifestations of trauma-related distress.

**Table 7:** Correlation matrix of composite scores.

Variable	1	2	3	4	5
Mindfulness	1				
Self compassion	0.42^***^	1			
PTSD symptoms	−0.27^***^	−0.28^***^	1		
Depressive symptoms	−0.42^***^	−0.35^***^	0.59^***^	1	
Dissociation	−0.26^***^	−0.25^***^	0.54^***^	0.59^***^	1

^***^Indicates statistical significance at the *p* < 0.001 level.

### 4.4 Regression analysis of mindfulness and self-compassion on trauma symptoms

Table 8 reports the regression results for PTSD symptoms. Both mindfulness and self-compassion emerged as significant negative predictors of PTSD symptoms. Specifically, mindfulness was significantly associated with lower PTSD symptoms (*B* = −0.226, SE = 0.082, β = −0.187, *t* = −2.756, *p* = 0.006), indicating that participants with higher levels of mindfulness tended to report fewer PTSD symptoms. Similarly, self-compassion also showed a significant negative effect on PTSD symptoms (*B* = −0.228, SE = 0.084, β = −0.184, *t* = −2.709, *p* = 0.007), suggesting that greater self-compassion was likewise related to lower post-traumatic stress symptomatology. The standardized coefficients were similar in magnitude, indicating that mindfulness and self-compassion made comparable contributions to explaining PTSD symptoms in the model. Overall, the regression model was statistically significant, *F* = 12.902, *p* < 0.001, although the explained variance was modest (*R*^2^ = 0.098, adjusted *R*^2^ = 0.091). This suggests that while mindfulness and self-compassion were both meaningful protective factors, a substantial proportion of the variance in PTSD symptoms was likely attributable to other unmeasured factors.

**Table 8:** Regression analysis predicting PTSD symptoms.

Predictor	*B*	SE	β	*T*	*p*	Sig.
Constant	5.181	0.382		13.567	< 0.001	^***^
Mindfulness	−0.226	0.082	−0.187	−2.756	0.006	^**^
Self compassion	−0.228	0.084	−0.184	−2.709	0.007	^**^
*R* ^2^	0.098					
Adjusted *R*^2^	0.091					
*F*	12.902				< 0.001	^***^

^***^Indicates statistical significance at the *p* < 0.001 level; ^**^Indicates statistical significance at the *p* < 0.01 level.

Table 9 presents the regression results for depressive symptoms. Both mindfulness and self-compassion significantly and negatively predicted depressive symptoms. Specifically, mindfulness showed a significant negative association with depressive symptoms (*B* = −0.416, SE = 0.080, β = −0.330, *t* = −5.191, *p* < 0.001), indicating that participants with higher levels of mindfulness tended to report lower levels of depressive symptoms. Self-compassion also emerged as a significant negative predictor (*B* = −0.268, SE = 0.082, β = −0.207, *t* = −3.254, *p* = 0.001), suggesting that greater self-compassion was likewise associated with fewer depressive symptoms. Compared with self-compassion, mindfulness showed a somewhat stronger standardized effect, indicating that mindfulness contributed more strongly to the reduction of depressive symptoms in this model. The overall regression model was statistically significant, *F* = 31.476, *p* < 0.001, and explained 21.0% of the variance in depressive symptoms (*R*^2^ = 0.210, adjusted *R*^2^ = 0.203). This proportion was notably higher than that observed in the PTSD model, suggesting that mindfulness and self-compassion may be more strongly related to depressive symptoms than to PTSD symptoms in the present sample.

**Table 9:** Regression analysis predicting depressive symptoms.

Predictor	*B*	SE	β	*t*	*p*	Sig.
Constant	6.545	0.373		17.524	< 0.001	^***^
Mindfulness	−0.416	0.080	−0.330	−5.191	< 0.001	^***^
Self Compassion	−0.268	0.082	−0.207	−3.254	0.001	^**^
*R* ^2^	0.210					
Adjusted *R*^2^	0.203					
*F*	31.476				< 0.001	^***^

^***^Indicates statistical significance at the *p* < 0.001 level; ^**^Indicates statistical significance at the *p* < 0.01 level.

Table 10 reports the regression results for dissociation. Both mindfulness and self-compassion significantly and negatively predicted dissociation. Specifically, mindfulness was negatively associated with dissociation (*B* = −0.223, SE = 0.082, β = −0.185, *t* = −2.710, *p* = 0.007), indicating that higher levels of mindfulness were associated with lower levels of dissociative symptoms. Self-compassion also showed a significant negative effect on dissociation (*B* = −0.212, SE = 0.084, β = −0.171, *t* = −2.509, *p* = 0.013), suggesting that participants with greater self-compassion likewise tended to report less dissociation. The standardized coefficients indicate that the effects of mindfulness and self-compassion were relatively similar in magnitude, although the effect of mindfulness was slightly stronger. The overall regression model was statistically significant, *F* = 11.778, *p* < 0.001, but the amount of explained variance was modest (*R*^2^ = 0.090, adjusted *R*^2^ = 0.083). This suggests that mindfulness and self-compassion were both meaningful protective correlates of dissociation, although dissociative symptoms were likely also shaped by other factors not included in the present model.

**Table 10:** Regression analysis predicting dissociation.

Predictor	*B*	SE	β	*t*	*p*	Sig.
Constant	5.707	0.383		14.910	< 0.001	^***^
Mindfulness	−0.223	0.082	−0.185	−2.710	0.007	^**^
Self compassion	−0.212	0.084	−0.171	−2.509	0.013	^*^
*R* ^2^	0.090					
Adjusted *R*^2^	0.083					
*F*	11.778				< 0.001	^***^

^***^Indicates statistical significance at the *p* < 0.001 level; ^**^Indicates statistical significance at the *p* < 0.01 level; ^*^Indicates statistical significance at the *p* < 0.05 level.

Table 11 presents the regression results for overall trauma symptoms. Both mindfulness and self-compassion significantly and negatively predicted trauma symptoms. Specifically, mindfulness showed a significant negative association with overall trauma symptoms (*B* = −0.288, SE = 0.066, β = −0.282, *t* = −4.368, *p* < 0.001), indicating that participants with higher levels of mindfulness tended to report lower levels of trauma-related symptoms overall. Self-compassion also emerged as a significant negative predictor (*B* = −0.236, SE = 0.068, β = −0.225, *t* = −3.481, *p* < 0.001), suggesting that greater self-compassion was similarly associated with reduced trauma symptomatology. Compared with self-compassion, mindfulness showed a somewhat stronger standardized effect, although both predictors made meaningful independent contributions to the model. The overall regression model was statistically significant, *F* = 26.762, *p* < 0.001, and explained 18.4% of the variance in trauma symptoms (*R*^2^ = 0.184, adjusted *R*^2^ = 0.177). These findings indicate that mindfulness and self-compassion functioned as important protective psychological resources in relation to overall trauma-related distress.

**Table 11:** Regression analysis predicting trauma symptoms.

Predictor	*B*	SE	β	*t*	*P*	Sig.
Constant	5.811	0.307		18.905	< 0.001	^***^
Mindfulness	−0.288	0.066	−0.282	−4.368	< 0.001	^***^
Self compassion	−0.236	0.068	−0.225	−3.481	< 0.001	^***^
*R* ^2^	0.184					
Adjusted *R*^2^	0.177					
*F*	26.762				< 0.001	^***^

^***^Indicates statistical significance at the *p* < 0.001 level; ^*^Indicates statistical significance at the *p* < 0.05 level.

### 4.5 Moderating effect of trauma severity

Table 12 presents the moderation analysis examining whether self-compassion moderated the association between trauma severity and PTSD symptoms. Trauma severity showed a strong and significant positive effect on PTSD symptoms (*B* = 0.893, SE = 0.048, β = 0.779, *t* = 18.496, *p* < 0.001), indicating that higher levels of trauma severity were associated with substantially higher levels of PTSD symptoms. In contrast, the main effect of self-compassion was not statistically significant (*B* = 0.016, SE = 0.052, β = 0.013, *t* = 0.316, *p* = 0.752), suggesting that self-compassion did not independently predict PTSD symptoms once trauma severity and the interaction term were included in the model. Importantly, the interaction between trauma severity and self-compassion was positive and statistically significant (*B* = 0.112, SE = 0.028, β = 0.155, *t* = 3.940, *p* < 0.001). This result indicates that the relationship between trauma severity and PTSD symptoms varied as a function of self-compassion. Specifically, as self-compassion increased, the positive association between trauma severity and PTSD symptoms became stronger rather than weaker. Therefore, the interaction pattern does not support a buffering effect of self-compassion in the present model. The overall regression model was statistically significant, *F* = 138.569, *p* < 0.001, and explained 63.8% of the variance in PTSD symptoms (*R*^2^ = 0.638, adjusted *R*^2^ = 0.633), suggesting that the model had substantial explanatory power.

**Table 12:** Moderation analysis of trauma severity × self compassion on PTSD symptoms.

Predictor	*B*	SE	β	*t*	*p*	Sig.
Constant	3.452	0.082		42.066	< 0.001	^***^
Trauma severity	0.893	0.048	0.779	18.496	< 0.001	^***^
Self compassion	0.016	0.052	0.013	0.316	0.752	
Trauma severity × self compassion	0.112	0.028	0.155	3.940	< 0.001	^***^
*R* ^2^	0.638					
Adjusted *R*^2^	0.633					
*F*	138.569				< 0.001	^***^

^***^Indicates statistical significance at the *p* < 0.001 level.

Table 13 presents the moderation analysis examining whether mindfulness moderated the association between trauma severity and PTSD symptoms. Trauma severity showed a strong and significant positive effect on PTSD symptoms (*B* = 0.918, SE = 0.049, β = 0.802, *t* = 18.657, *p* < 0.001), indicating that greater trauma severity was associated with higher levels of PTSD symptoms. The main effect of mindfulness was not statistically significant (*B* = 0.068, SE = 0.052, β = 0.056, *t* = 1.303, *p* = 0.194), suggesting that mindfulness did not independently predict PTSD symptoms once trauma severity and the interaction term were included in the model. Importantly, the interaction between trauma severity and mindfulness was positive and statistically significant (*B* = 0.094, SE = 0.030, β = 0.125, *t* = 3.143, *p* = 0.002). This indicates that mindfulness significantly moderated the relationship between trauma severity and PTSD symptoms. However, because the interaction coefficient was positive, the pattern does not support a buffering effect. Rather, the positive association between trauma severity and PTSD symptoms became stronger at higher levels of mindfulness. The overall model was statistically significant, *F* = 134.532, *p* < 0.001, and explained 63.1% of the variance in PTSD symptoms (*R*^2^ = 0.631, adjusted *R*^2^ = 0.626), indicating substantial explanatory power.

**Table 13:** Moderation analysis of trauma severity × mindfulness on PTSD symptoms.

Predictor	*B*	SE	β	*t*	*p*	Sig.
Constant	3.445	0.084		40.772	< 0.001	^***^
Trauma severity	0.918	0.049	0.802	18.657	< 0.001	^***^
Mindfulness	0.068	0.052	0.056	1.303	0.194	
Trauma severity × mindfulness	0.094	0.030	0.125	3.143	0.002	^**^
*R* ^2^	0.631					
Adjusted *R*^2^	0.626					
*F*	134.532				< 0.001	^***^

^***^Indicates statistical significance at the *p* < 0.001 level; ^**^Indicates statistical significance at the *p* < 0.01 level.

### 4.6 Regional differences

Table 14 presents the independent-samples *t*-test results for regional differences between the eastern and western groups. The findings showed that the eastern group reported a slightly higher mean level of mindfulness (*M* = 4.32, SD = 1.62) than the western group (*M* = 3.93, SD = 1.64), but this difference did not reach statistical significance (*t* = 1.835, *p* = 0.068). Similarly, no significant regional difference was found for self-compassion, with the eastern group (*M* = 4.01, SD = 1.55) and the western group (*M* = 3.96, SD = 1.65) showing highly comparable levels (*t* = 0.269, *p* = 0.788). In contrast, significant regional differences emerged for PTSD symptoms and overall trauma symptoms. Specifically, participants in the western group reported significantly higher PTSD symptoms (*M* = 3.71, SD = 2.00) than those in the eastern group (*M* = 2.98, SD = 1.89), *t* = −2.925, *p* = 0.004. Likewise, the western group showed significantly higher overall trauma symptoms (*M* = 3.94, SD = 1.65) than the eastern group (*M* = 3.43, SD = 1.66), *t* = −2.395, *p* = 0.017. Although the western group also had somewhat higher mean scores on depressive symptoms and dissociation, these differences were not statistically significant (*p* = 0.085 and *p* = 0.179, respectively). Overall, these results suggest that while mindfulness and self-compassion did not differ substantially across regions, trauma-related distress—particularly PTSD symptoms and overall trauma symptoms—was more pronounced in the western group.

**Table 14:** Independent-samples *t*-test for regional differences.

Variable	East M (SD)	West M (SD)	*t*	*p*	Sig.
Mindfulness	4.32 (1.62)	3.93 (1.64)	1.835	0.068	
Self compassion	4.01 (1.55)	3.96 (1.65)	0.269	0.788	
PTSD symptoms	2.98 (1.89)	3.71 (2.00)	−2.925	0.004	^**^
Depressive symptoms	3.53 (2.03)	3.99 (2.07)	−1.730	0.085	
Dissociation	3.77 (2.03)	4.12 (1.90)	−1.347	0.179	
Trauma symptoms	3.43 (1.66)	3.94 (1.65)	−2.395	0.017	^*^

^**^Indicates statistical significance at the p < 0.01 level; ^*^Indicates statistical significance at the *p* < 0.05 level.

### 4.7 Longitudinal predictive effects

Table 15 reports the longitudinal predictive effects of baseline mindfulness and self-compassion on subsequent trauma-related symptoms at T2. The results showed that mindfulness at T0 significantly and negatively predicted PTSD symptoms at T2 (*B* = −0.487, SE = 0.027, β = −0.766, *t* = −18.376, *p* < 0.001), depressive symptoms at T2 (*B* = −0.354, SE = 0.028, β = −0.640, *t* = −12.856, *p* < 0.001), and dissociation at T2 (*B* = −0.318, SE = 0.025, β = −0.641, *t* = −12.893, *p* < 0.001). These findings indicate that higher baseline mindfulness was consistently associated with lower levels of later trauma-related symptoms. Similarly, self-compassion at T0 also significantly and negatively predicted PTSD symptoms at T2 (*B* = −0.433, SE = 0.031, β = −0.677, *t* = −14.202, *p* < 0.001), depressive symptoms at T2 (*B* = −0.378, SE = 0.026, β = −0.680, *t* = −14.312, *p* < 0.001), and dissociation at T2 (*B* = −0.291, SE = 0.026, β = −0.583, *t* = −11.083, *p* < 0.001). Overall, all longitudinal effects were statistically significant and negative, suggesting that both mindfulness and self-compassion functioned as robust protective psychological resources over time. Comparatively, mindfulness showed the strongest predictive effect on later PTSD symptoms, whereas both mindfulness and self-compassion demonstrated similarly substantial negative associations with later depressive symptoms. Taken together, these results provide strong support for the longitudinal role of mindfulness and self-compassion in reducing subsequent trauma-related distress.

**Table 15:** Longitudinal predictive effects.

Predictor	Outcome	*B*	SE	β	*t*	*p*	Sig.
Mindfulness_T0	PTSD_T2	−0.487	0.027	−0.766	−18.376	< 0.001	^***^
Mindfulness_T0	Depressive Symptoms_T2	−0.354	0.028	−0.640	−12.856	< 0.001	^***^
Mindfulness_T0	Dissociation_T2	−0.318	0.025	−0.641	−12.893	< 0.001	^***^
Self Compassion_T0	PTSD_T2	−0.433	0.031	−0.677	−14.202	< 0.001	^***^
Self Compassion_T0	Depressive symptoms_T2	−0.378	0.026	−0.680	−14.312	< 0.001	^***^
Self Compassion_T0	Dissociation_T2	−0.291	0.026	−0.583	−11.083	< 0.001	^***^

^***^Indicates statistical significance at the *p* < 0.001 level.

## 5 Discussion

### 5.1 Mindfulness and self-compassion as protective psychological resources in trauma-related distress

The findings related to H1 and H2 suggest that mindfulness and self-compassion functioned as meaningful protective psychological resources in trauma-related distress among adult women, although their strength was not identical across symptom domains. H1 was supported by the correlational results. At the composite level, mindfulness was positively associated with self-compassion (*r* = 0.42, *p* < 0.001) and negatively associated with PTSD symptoms (*r* = −0.27, *p* < 0.001), depressive symptoms (*r* = −0.42, *p* < 0.001), and dissociation (*r* = −0.26, *p* < 0.001), while self-compassion showed similarly negative associations with PTSD symptoms (*r* = −0.28, *p* < 0.001), depressive symptoms (*r* = −0.35, *p* < 0.001), and dissociation (*r* = −0.25, *p* < 0.001) (Table 7). The same overall pattern also appeared at the indicator level, where observation, acting with awareness, non-reactivity, self-kindness, common humanity, and reduced self-criticism were all negatively related to trauma-related symptoms (Table 6). These findings are consistent with the everyday reality of trauma-exposed women, whose suffering often involves not only intrusive stress responses but also shame, sadness, emotional exhaustion, and disrupted self-relationship. In such contexts, present-moment awareness and a less self-critical stance may plausibly reduce emotional entanglement and psychological burden. H2 was also supported, as mindfulness and self-compassion independently predicted lower trauma-related symptoms across the regression models. Both were significant negative predictors of PTSD symptoms (Table 8), depressive symptoms (Table 9), dissociation (Table 10), and overall trauma symptoms (Table 11). Notably, their effects were stronger for depressive symptoms and overall trauma symptoms than for PTSD symptoms and dissociation. For example, mindfulness (*B* = −0.416, β = −0.330, *p* < 0.001) and self-compassion (*B* = −0.268, β = −0.207, *p* = 0.001) explained 21.0% of the variance in depressive symptoms (Table 9), whereas the PTSD model explained 9.8% (Table 8). This suggests that mindfulness and self-compassion may be especially relevant to broader affective suffering and generalized trauma burden rather than equally powerful across all trauma-specific reactions. Overall, these findings support the view that mindfulness and self-compassion are not peripheral traits, but psychologically meaningful resources associated with lower trauma-related distress in adult women.

### 5.2 Longitudinal predictive role of mindfulness and self-compassion in women's trauma adjustment

The results related to H3 indicate that mindfulness and self-compassion were not merely concurrent correlates of trauma-related distress, but also showed clear prospective relevance for later symptom levels. Baseline mindfulness significantly and negatively predicted PTSD symptoms at T2 (*B* = −0.487, β = −0.766, *p* < 0.001), depressive symptoms at T2 (*B* = −0.354, β = −0.640, *p* < 0.001), and dissociation at T2 (*B* = −0.318, β = −0.641, *p* < 0.001). Baseline self-compassion showed the same overall pattern, significantly predicting lower PTSD symptoms (*B* = −0.433, β = −0.677, *p* < 0.001), depressive symptoms (*B* = −0.378, β = −0.680, *p* < 0.001), and dissociation (*B* = −0.291, β = −0.583, *p* < 0.001) at T2 (Table 15). These findings suggest that women who entered the study with greater present-moment awareness, less reactivity, more self-kindness, and less self-criticism were more likely to report lower trauma-related distress several months later. This pattern is important because trauma recovery in women is rarely immediate or linear. In many real-life settings, women continue to live under ongoing interpersonal, economic, or caregiving pressures even after the traumatic event itself, meaning that recovery depends not only on the absence of stress but also on the availability of inner resources that shape how distress is processed over time. From this perspective, mindfulness may help by reducing experiential avoidance and promoting more stable emotional regulation, whereas self-compassion may reduce shame, self-blame, and harsh self-evaluation, all of which are common barriers to recovery in trauma-exposed women. The especially strong longitudinal associations observed for later PTSD and depressive symptoms suggest that these two resources may be particularly relevant to the persistence or attenuation of emotional suffering over time. At the same time, these results should still be interpreted cautiously as longitudinal predictive associations rather than definitive causal effects, because later symptom levels are also likely influenced by many unmeasured contextual factors, such as social support, safety, service access, and cumulative stress. Even so, the consistency of the findings across all three symptom domains indicates that mindfulness and self-compassion may have practical value not only as correlates of current well-being but also as resources relevant to longer-term post-trauma adjustment in adult women.

### 5.3 Regional variation and the context-dependent nature of trauma recovery

The findings related to H4 suggest that trauma recovery in adult women is shaped not only by individual psychological resources but also by the broader context in which those resources operate. H4 received partial support. Mindfulness and self-compassion did not differ significantly between the eastern and western groups, although the eastern group reported slightly higher mindfulness (*M* = 4.32, SD = 1.62) than the western group (*M* = 3.93, SD = 1.64), and slightly higher self-compassion (*M* = 4.01, SD = 1.55 vs. 3.96, SD = 1.65); however, neither difference reached statistical significance (Table 14). In contrast, clear regional differences emerged for trauma-related symptoms. Women in the western group reported significantly higher PTSD symptoms (*M* = 3.71, SD = 2.00) than those in the eastern group (*M* = 2.98, SD = 1.89, *p* = 0.004), and they also showed significantly higher overall trauma symptoms (*M* = 3.94, SD = 1.65 vs. 3.43, SD = 1.66, *p* = 0.017). Although depressive symptoms and dissociation were also higher in the western group, these differences did not reach significance. This pattern suggests that regional variation in the present study was more evident in symptom burden than in the average level of psychological resources. Such a result is highly plausible in real-world settings, because women's trauma-related distress is often shaped not only by internal coping capacities but also by differences in service access, social support, economic insecurity, caregiving pressure, stigma, and cumulative adversity. In other words, women across regions may possess broadly similar levels of mindfulness and self-compassion, yet still experience different levels of trauma-related suffering because the environments in which recovery takes place are not equivalent. The moderation results further reinforce the importance of context. Although trauma severity strongly predicted PTSD symptoms, the interaction terms Trauma Severity × Self-Compassion (*B* = 0.112, β = 0.155, *p* < 0.001) and Trauma Severity × Mindfulness (*B* = 0.094, β = 0.125, *p* = 0.002) were both positive (Tables 12 and 13), indicating that the relationship between trauma severity and PTSD symptoms became stronger rather than weaker at higher levels of these resources. Thus, the data do not support a simple buffering interpretation. Instead, they suggest that the role of mindfulness and self-compassion may depend on the severity of trauma exposure and the surrounding context. Under more severe trauma conditions, individual resources alone may be insufficient to offset symptom escalation, and greater awareness or self-reflection may even coincide with more conscious recognition of distress. Taken together, these findings indicate that trauma recovery in women is context-dependent: mindfulness and self-compassion appear meaningful, but their role is embedded within broader regional and situational realities rather than operating as universally protective mechanisms.

## 6 Conclusion

The present study provides evidence that mindfulness and self-compassion are meaningful psychological resources associated with lower trauma-related distress among trauma-exposed adult women. Across the correlational and regression analyses, higher levels of mindfulness and self-compassion were linked to lower PTSD symptoms, depressive symptoms, dissociation, and overall trauma symptoms, indicating that these resources are relevant not only to a single trauma outcome but to women's broader post-trauma psychological adjustment. The longitudinal results further strengthen this interpretation by showing that baseline mindfulness and self-compassion predicted lower levels of later trauma-related symptoms, suggesting that their importance extends beyond concurrent associations and may be relevant to longer-term adjustment processes. At the same time, the findings indicate that trauma recovery is context-dependent rather than uniformly determined by internal resources alone. Regional differences were more evident in symptom burden than in the average level of mindfulness and self-compassion, and the moderation analyses did not support a simple buffering interpretation under higher trauma severity. Taken together, these results suggest that mindfulness and self-compassion should be understood as important but not sufficient protective resources. Their value in trauma-informed support for adult women is likely greatest when they are integrated with broader psychosocial support, context-sensitive services, and interventions responsive to the severity and complexity of women's trauma-related experiences.

## 7 Limitations

First, although the study employed a three-wave design, the principal longitudinal analyses primarily examined associations between baseline variables (T0) and later outcomes at T2. As a result, the intermediate T1 assessment was not fully incorporated into the main analytical models. Future research may benefit from using all three waves more comprehensively, such as through cross-lagged or growth-trajectory approaches, in order to better capture dynamic changes in mindfulness, self-compassion, and trauma-related symptoms over time.

## Data Availability

The raw data supporting the conclusions of this article will be made available by the authors, without undue reservation.
